# Structure and Antitumor and Immunomodulatory Activities of a Water-Soluble Polysaccharide from *Dimocarpus longan* Pulp

**DOI:** 10.3390/ijms15035140

**Published:** 2014-03-24

**Authors:** Fa-Yan Meng, Yuan-Ling Ning, Jia Qi, Zhou He, Jiang Jie, Juan-Juan Lin, Yan-Jun Huang, Fu-Sen Li, Xue-Hua Li

**Affiliations:** 1 School of Pharmaceutical Sciences, Guangxi Medical University, No. 22 Shuangyong Road, Nanning 530021, Guangxi, China; E-Mails: fayanmeng@gmail.com (F.-Y.M.); jiejiang8084@gmail.com (J.J.); linjuanjuan8086@gmail.com (J.-J.L.); yanjunhuang69@gmail.com (Y.-J.H.); onlythinkforyou@sina.com (F.-S.L.); 2 Wu Jieping Medical Foundation Cell and Molecular Clinical Research Center, Beihai People’s Hospital, No. 83 Heping Road, Beihai 536000, Guangxi, China; E-Mail: ningyuanling@gmail.com; 3 Department of Pharmacy, Heilongjiang Nursing College, No. 209 Xuefu Road, Harbin 150036, Heilongjiang, China; E-Mail: jiaqi1a2@gmail.com; 4 Department of Acupuncture and Moxibustion, the People’s Hospital of Guangxi Zhuang Autonomous Region, No. 6 Taoyuan Road, Nanning 530021, Guangxi, China; E-Mail: hezhou461@gmail.com

**Keywords:** structure, antitumor activity, immunomodulatory activity, polysaccharide, *Dimocarpus longan* pulp

## Abstract

A new water-soluble polysaccharide (longan polysaccharide 1 (LP1)) was extracted and successfully purified from *Dimocarpus longan* pulp via diethylaminoethyl (DEAE)-cellulose anion-exchange and Sephacryl S-300 HR gel chromatography. The chemical structure was determined using Infrared (IR), gas chromatography (GC) and nuclear magnetic resonance (NMR) analysis. The results indicated that the molecular weight of the sample was 1.1 × 10^5^ Da. Monosaccharide composition analysis revealed that LP1 was composed of Glc, GalA, Ara and Gal in a molar ratio of 5.39:1.04:0.74:0.21. Structural analysis indicated that LP1 consisted of a backbone of →4)-α-d-Glc*p*-(1→4)-α-d-Gal*p*A-(1→4)-α-d-Glc*p*-(1→4)-β-d-Glc*p*-(1→ units with poly saccharide side chains composed of →2)-β-d-Fruf-(1→2)-l-sorbose-(1→ attached to the O-6 position of the α-d-Glc*p* residues. *In vitro* experiments indicated that LP1 had significantly high antitumor activity against SKOV3 and HO8910 tumor cells, with inhibition percentages of 40% and 50%, respectively. In addition, LP1 significantly stimulated the production of the cytokine interferon-γ (IFN-γ), increased the activity of murine macrophages and enhanced B- and T-lymphocyte proliferation. The results of this study demonstrate that LP1 has potential applications as a natural antitumor agent with immunomodulatory activity.

## 1. Introduction

*Dimocarpus longan* pulp (longan) is a commercially available fruit widely distributed in southern China. Longan pulp has long been used in China to promote health and blood metabolism, soothe nerves, prevent amnesia, relieve insomnia and extend longevity [1,2]. Several recent studies have revealed that the alcohol extracts of longan pulp reduce serum prolactin levels in female rats [3]. The water extracts of longan pulp show a measurable effect against the JTC26 cervical cancer cell line, as confirmed by Cai *et al.* [4]. In addition, longan pulp polysaccharides have demonstrated immunomodulatory activity, as reported by Chen *et al.* [5].

Polysaccharides, which are widely distributed in fruit, animals, plants and fungi, have drawn increasing attention from researchers and consumers, due to their obvious antitumor, antioxidant [6], anti-HIV/AIDS and immunostimulatory activities [7,8], as well as the relatively low toxicity [9] of the polysaccharides. Therefore, the discovery and evaluation of polysaccharides with antitumor and immunostimulatory properties has become an important focus of research in chemistry and biology [10].

However, information regarding the polysaccharides from longan pulp and the *in vitro* immunomodulatory and antitumor properties of longan polysaccharides (LPs) is limited. Therefore, as reported in this paper, a purified fraction, referred to as LP1, was obtained from crude polysaccharide extract from longan pulp via diethylaminoethyl (DEAE)-cellulose anion-exchange and Sephacryl S-300 HR gel chromatography. The chemical structure of the polysaccharide and its antitumor activity *in vitro* and immunomodulatory activity *in vivo* were investigated.

## 2. Results and Discussion

### 2.1. Isolation, Purification and Molecular Weight of the Polysaccharide

**Figure 1.**
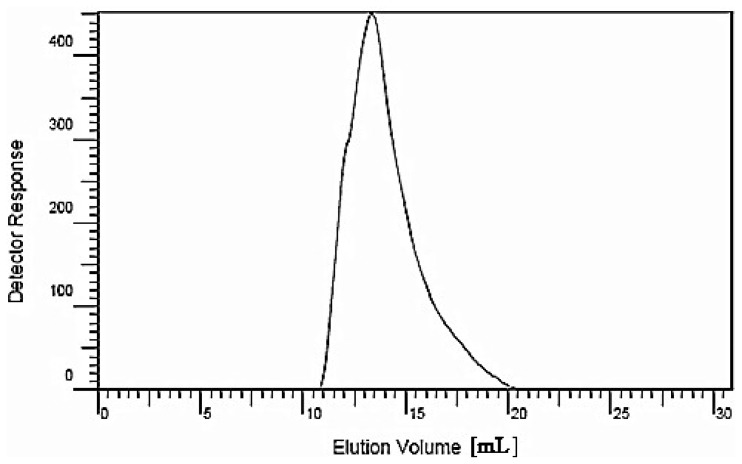
Determination of the molecular weight (*M*_r_) of longan polysaccharide 1 (LP1) via high-performance gel permeation chromatography (HPGPC).

**Figure 2.**
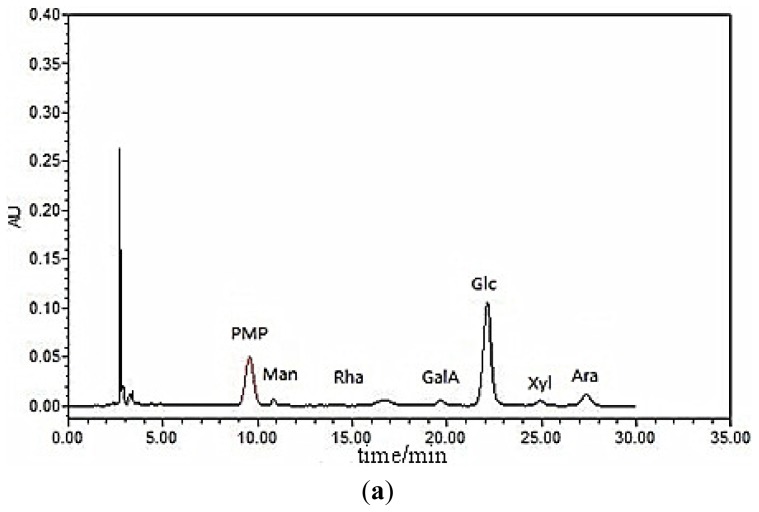

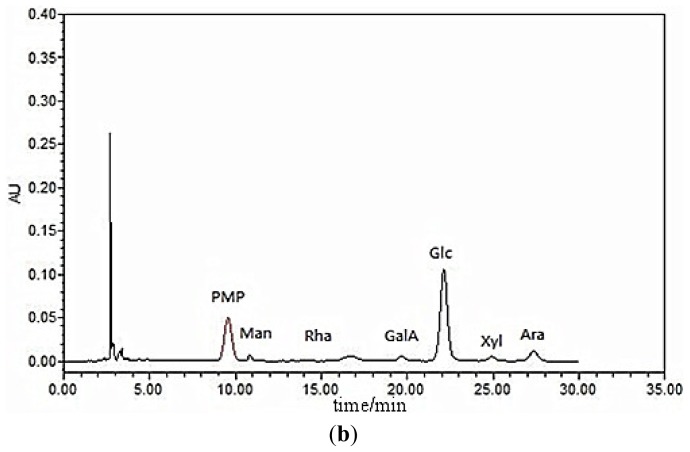
(**a**) High-performance liquid chromatography (HPLC) of standard monosaccharides; and (**b**) HPLC of LP1 after complete acid hydrolysis. PMP, 1-phenyl-3-methyl-5-pyrazolone.

A crude polysaccharide from longan pulp was obtained via hot water extraction, alcohol precipitation and deproteinization. After successive separation with DEAE-cellulose anion-exchange and Sephacryl S-300 high resolution (HR) gel filtration chromatography, a water-soluble polysaccharide (LP1) was obtained. LP1 showed only one single symmetric peak on high-performance gel permeation chromatography (HPGPC), implying that LP1 is a homogeneous polysaccharide (Figure 1). No absorption was observed at 280 nm, which suggested that LP1 did not contain protein. LP1 was hydrolyzed with trifluoroacetic acid into individual monosaccharides that were trimethylsilylated for high-performance liquid chromatography (HPLC) analysis (Figure 2a). By comparing the retention times with the standard monosaccharides, the monosaccharide composition was identified (Figure 2b). Six monosaccharides, including Man, Rha, GalA, Glc, Gal and Ara, were identified. The molar ratio of the major monosaccharides, Glc, GalA, Ara and Gal, was found to be 5.39:1.04:0.74:0.21. The results suggested that Glc constituted the backbone of LP1 in combination with GalA, Ara and Gal.

The average molecular weight (*M*_r_) of LP1 was estimated by using standard dextrans as a reference. From the standard curve, we obtained a linear regression equation of log *M*_r_ = −0.4073 *V*_e_ + 8.4461 (*V*_e_ = evolution volume), *r*^2^ = 0.9972, and the *M*_r_ of the homogeneous polysaccharide, LP1, was determined to be 1.1 × 10^5^ Da.

### 2.2. Chemical Structure

#### 2.2.1. Fourier Transform Infrared (FT-IR) Spectrum Characterization

**Figure 3.**
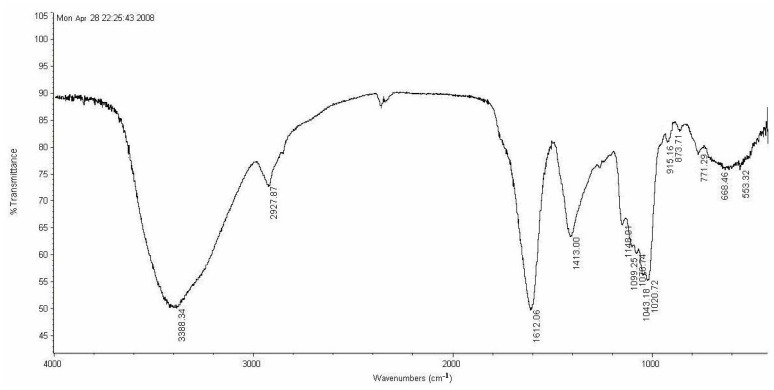
Fourier transform infrared (FT-IR) spectrum of LP1.

The infrared spectrum of LP1 from 400–4000 cm^−1^ is shown in Figure 3. A broad stretching vibration peak was observed at approximately 3388 cm^−1^, corresponding to a hydroxy group, and this peak also indicates that an intramolecular hydrogen bond exists within the polysaccharide. A strong C–H asymmetric stretching vibration peak was observed at 2927 cm^−1^. An asymmetric stretching vibration peak at 1612 cm^−1^ indicates the presence of a deprotonated carboxylic group (COO^−^), which is similar to the characteristics of polysaccharides reported by Zhang *et al.* [9]. A C–H bending vibration peak was observed at 1413 cm^−1^, and a strong absorption peak was detected at 1078 cm^−1^, which corresponds to pyranoside [11]. The range of 1200–1000 cm^−1^ is of special interest, because the vibration of the ring, the C–O–H stretching vibration and the pyran ring C–O–C and C–O stretching vibrations are superimposed in this area. The characteristic absorption band for a β-linked pyranose was observed at 873 cm^−1^ [12]. Bands characteristic of polysaccharides containing a β-type pyran ring were observed in the spectrum.

#### 2.2.2. NMR Spectroscopic Analysis

**Figure 4.**
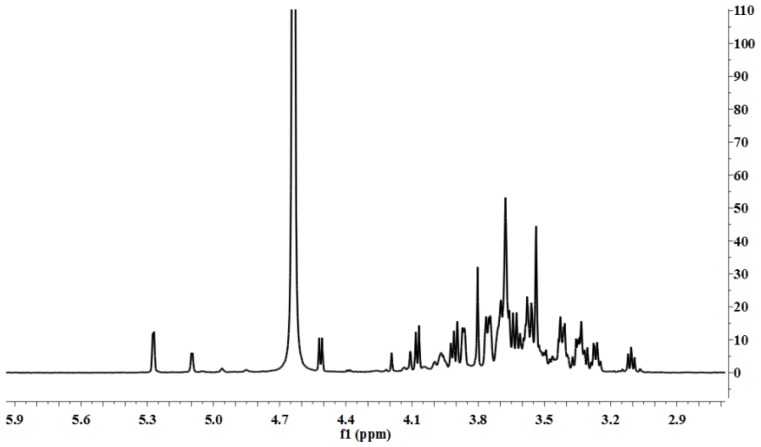
^1^H NMR spectrum of LP1.

**Figure 5.**
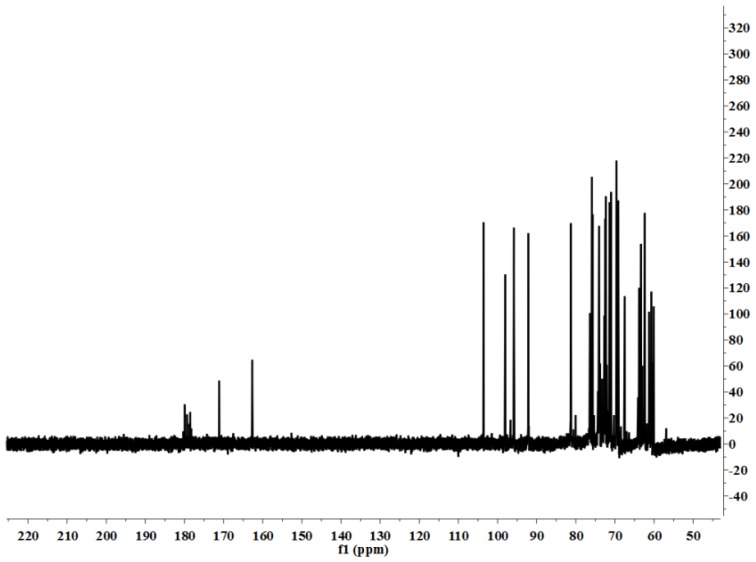
^13^C NMR spectrum of LP1.

**Figure 6.**
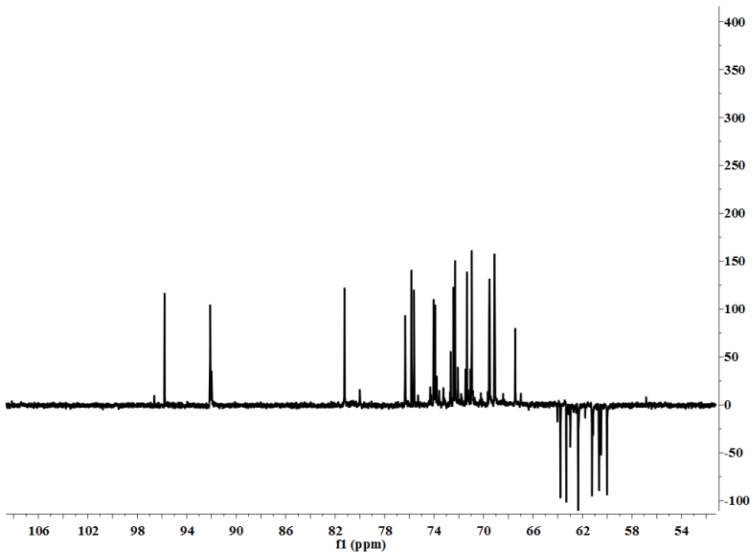
DEPT135 spectrum of LP1.

**Figure 7.**
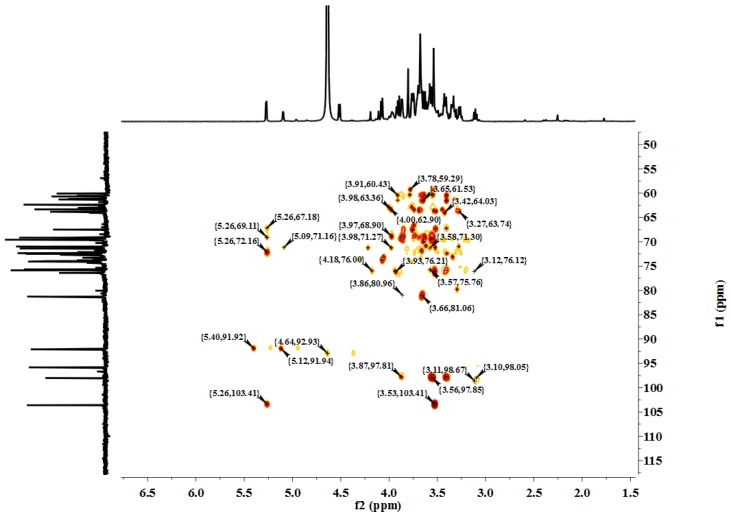
HMBC spectrum of LP1.

Figure 4 shows the ^1^H NMR spectrum of LP1. The anomeric ^1^H signals occurred at 5.27 and 5.09 ppm; the presence of these signals in the range of 4.91–5.34 ppm indicates that the configurations of the LP1 pyranose residues were primarily the α form [13]. The chemical shift of 4.51 ppm was the anomeric hydrogen of a β-pyranose [14]. Figure 5 shows the ^13^C NMR spectrum (125 MHz, 22 °C, dimethyl sulfoxide (DMSO)-*d*_6_). The chemical shifts at 103.60, 98.03, 95.82 and 92.13 ppm are attributed to the anomeric carbons of the sugar ring, and the shift at 81.25 ppm suggests the presence of (1→3/4) glycosidic linkages. The ^13^C NMR chemical shifts at 103.60 and 98.03 ppm did not appear in the DEPT135 spectrum (Figure 6), indicating that these two signals corresponded to quaternary carbon atoms. The signal at 103.60 ppm corresponded to the anomeric carbon of fructose, and the signal at 98.03 ppm corresponded to the anomeric carbon of sorbose. At 65.00–90.00 ppm, the signal is positive, indicating the methine carbon in the sugar ring; at 60.00–65.00 ppm, the signal is negative, corresponding to the methylene in the sugar ring [15–17]. The signal at 179.93 ppm results from the C-6 of galacturonic acid, and the signal at 71.11 ppm results from the C-4 of galacturonic acid. The C and H atom signals above coincide with the HMBC signals (Figure 7). Table 1 presents the ^1^H NMR and ^13^C NMR chemical shifts and their assignments. Table 2 shows the HMBC data and assignments for LP1.

From the ^1^H, ^13^C and heteronuclear multiple-bond correlation spectroscopy (HMBC) NMR data, we can deduce that LP1 is composed of fragments, as shown below:

**Figure:**
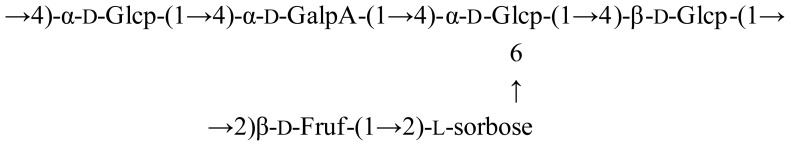


### 2.3. Activity

#### 2.3.1. *In Vitro* Inhibition of Tumor Cell Proliferation

Many chemical compounds are cytotoxic against cancer cells, but also toxic to normal cells [18,19]. In contrast, polysaccharides extracted from plants, fungi, algae and animals have shown fewer side effects when used as antitumor agents [20–25].

The percent inhibitions of LP1 at different doses against the SKOV3 and HO8910 tumor cells are summarized in Tables 3 and 4. Within a range of 5–40 mg/L, LP1 had obvious antitumor activity against SKOV3 tumor cells with inhibition percentages of 36.9% to 39.9%. Similarly, LP1 was also cytotoxic against HO8910 tumor cells; when the dose was increased to 320 mg/L, the inhibition percentage reached 50.3%. As shown in Tables 3 and 4, LP1 showed cytotoxicity in both HO8910 and SKOV3 tumor cells, and LP1 inhibited HO8910 cells to a greater extent compared to SKOV3 cells. The inhibition was dose-dependent for each cell line.

#### 2.3.2. Effect of LP1 on Macrophage Activity *in Vitro*

Macrophages are a type of antigen-presenting cell, and they differentiate and develop from bone marrow hematopoietic stem cells. Macrophages not only initiate innate immune responses, but also, they provide a defense mechanism against tumor cells [26–28]; these immune cells may simultaneously transfer the signal to lymphocytes and activate the acquired immune response. Activated macrophages can produce abundant reactive oxygen and nitrogen species *in vivo* [29], and these species may be capable of not only killing microorganisms, but also inhibiting cancer cells *in vivo* [30–32]. Therefore, the effect of longan pulp polysaccharide on macrophages was studied as a primary indicator of the effect of the polysaccharide on the immune system.

In this study, the phagocytic ability of macrophages was tested (Table 5), and the NO content of the supernatant (Table 6) was measured. The results indicate that LP1 significantly improves the phagocytic ability of macrophages and increases NO production.

Additionally, activated macrophages may also secrete a large number of cytokines, such as TNF-α, IL-6 and IL-1β, which have very important roles in the immune response of an organism [33]. IL-1β plays a key role in the network of cytokines that induce the activation of T-cells in the immune response. TNF-α is an important tumor necrosis factor secreted by macrophages [34]. IL-6 has a vital function in the immune response; most importantly, this cytokine induces mature CD4^+^ T-cells to differentiate to Th2 cells and promotes the secretion of immunoglobulin and acute phase globulin by B-cells [35–37]. To assess cytokine secretion related to the antitumor immunity induced by LP1, cytokine levels (TNF-α, IL-6 and IL-1β) were measured (Tables 7–9). Compared with the normal control group, LP1 significantly induced the secretion of TNF-α, IL-6 and IL-1β (*p* < 0.05). This result demonstrates that LP1 enhances immune function by inducing macrophages to secrete cytokines.

The experiments on the effect of LP1 on the proliferation of macrophages *in vitro* indicated that LP1 significantly enhanced the production of TNF-α, IL-6 and IL-1β and the release of NO, which suggests that the polysaccharide induced the functional activation of macrophages.

#### 2.3.3. Immunomodulatory Activities *in Vivo*

##### 2.3.3.1. Effect of LP1 on the Stimulation of Cytokine Production

**Figure 8.**
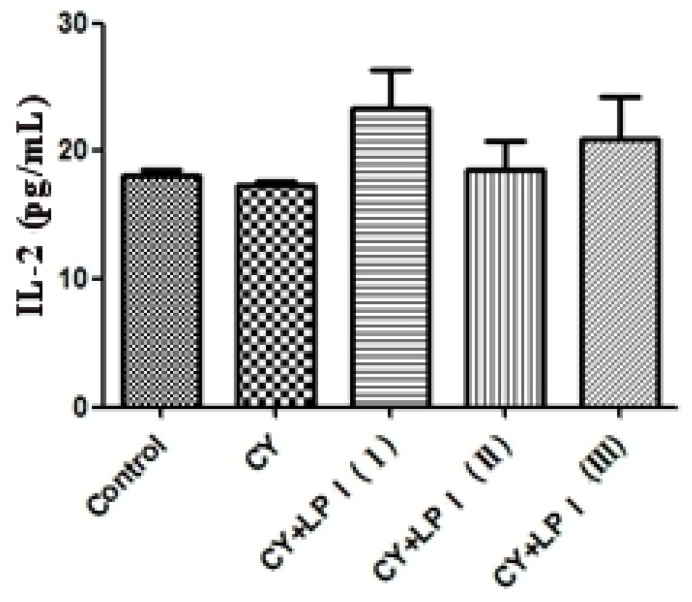
Effect of the LP1 polysaccharide from *Dimocarpus longan* pulp on the cyclophosphamide (CY)-induced immunosuppression of serum IL-2 levels in mice (*χ̄* ± SD, *n* = 8).

**Figure 9.**
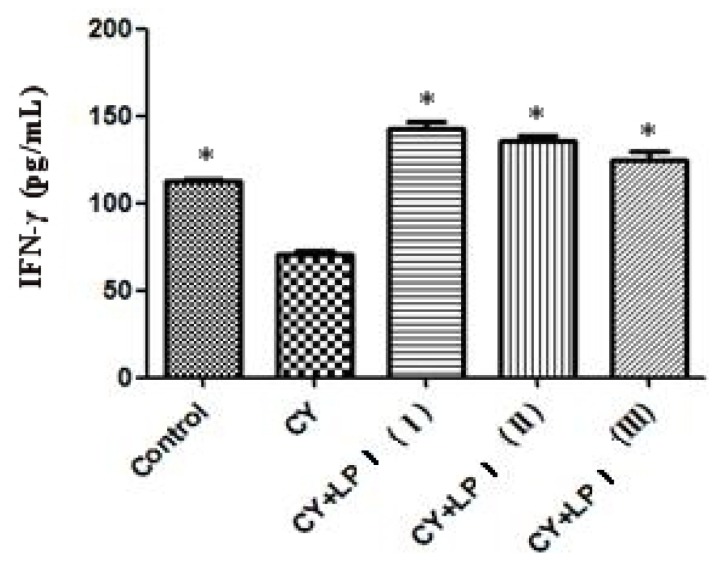
Effect of the LP1 polysaccharide from *Dimocarpus longan* pulp on cyclophosphamide (CY)-induced immunosuppression of serum IFN-γ levels in mice (*χ̄* ± SD, *n* = 8) (* compared with CY, *p* < 0.05).

Cytokines released by helper T-lymphocytes during the immune response play an important role in regulating the nature of the response. For example, interferon-γ (IFN-γ) and interleukin-2 (IL-2) are secreted by type 1 helper T-cells (Th1 cells) and mediate cellular immunity [38]. IL-2 is a type of T-cell growth factor; this cytokine stimulates the reactivity of many types of killer cells, promotes the expression of the insulin and transferrin receptors and generates several cytokines [39]. IFN-γ is a pleiotropic cytokine with immunomodulatory effects on different types of immune cells. In mammals, IFN-γ has been shown to be an indicator of cell-mediated immunity in infected organisms [40]. Therefore, a preliminary assessment of the extent of T-cell activation can be achieved by detecting the IFN-γ levels [41]. The effects of LP1 on the production of interleukin-2 and IFN-γ are shown in Figure 8. With LP1 stimulation, the production of IL-2 was only slightly higher than that obtained with cyclophosphamide (CY) treatment, but the production of IFN-γ after stimulation with LP1 was significantly higher than that with CY (*p* < 0.05) (Figure 9). A dose-dependent relationship was observed, and a dose of 320 mg/kg resulted in the highest IFN-γ production. The modulation of IFN-γ production may contribute to some of the therapeutic effects of LP1. These results indicate that LP1 may promote the secretion of IFN-γ by T-cells and T-cell proliferation, as well as Th1-mediated cellular immunity and the differentiation of Th1 cells. LP1 thus enhances Th1-mediated cellular immunity, thus enhancing adaptive immunity [7,39].

##### 2.3.3.2. Effects on Macrophage Phagocytosis

**Figure 10.**
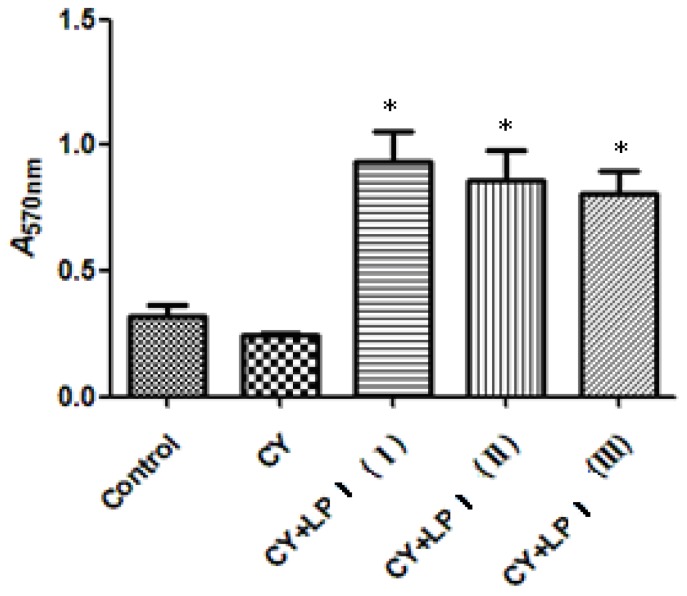
The effects of the LP1 polysaccharide from *Dimocarpus longan* pulp on cyclophosphamide (CY)-induced immunosuppression of the phagocytic ability of peritoneal macrophages as measured with neutral red (*χ̄* ± SD, *n* = 8) (* compared with CY alone, *p* < 0.05).

Macrophages are the most important professional phagocytes, which play a pivotal role in the host defense against any type of invading cell, including tumor cells [42]. Phagocytosis is a crucial defense mechanism for protection against pathogenic invasion in mammals [43]. In this study, the effects of LP1 at different concentrations were investigated using the neutral red method. The effect of LP1 on the phagocytosis of macrophages is depicted in Figure 10. The data indicate that, in the dose range of 80–320 mg/kg, each group had a higher absorbance than the CY group (*p* < 0.05), and the inducing effects were dose-dependent. A recent study proposed that arabinose, mannose, xylose and galactose played an important role in the stimulation of macrophages, but not glucose [44]. In this study, the enhancement of macrophage phagocytosis by LP1 might be mainly caused by the actions of the arabinose and galactose units of LP1. After phagocytosis, macrophages turn to their role as antigen-presenting cells, with the expression of higher levels of costimulatory molecules that mediate an interaction between T-cells and macrophages [45].

##### 2.3.3.3. Effects of LP1 on Spleen Lymphocyte Proliferation

T- and B-lymphocytes are two important classes of immunologically active cells and play important roles in enhancing the immune function of organisms [46]. The former is mainly responsible for cellular immunity and the latter is responsible for humoral immunity [47]. Spleen lymphocyte proliferation induced by ConA *in vitro* has been used to evaluate T-lymphocyte activity, while that induced by lipopolysaccharide (LPS) has been used to examine B-lymphocyte activity [48]. In this study, LP1 was evaluated by testing its effect on lymphocyte proliferation. As shown in Table 10, all the tested doses of LP1 did not significantly enhance splenocyte proliferation relative to CY (*p* > 0.05). However, in a synergistic stimulation with LPS, the *A*_570_ values for all the tested doses were significantly increased compared with the CY group (*p* < 0.05), which indicates that, with LPS-induced proliferation, LP1 significantly increases cell proliferation. The effects were dose-dependent in the range of 80–320 mg/kg.

Similarly, in the presence of ConA as a mitogen for lymphocytes, lymphocyte proliferation was studied, and the results are depicted in Table 11. The *A*_570_ values for all the tested doses were significantly increased compared with the CY group (*p* < 0.05); the proliferation of T-lymphocytes was also dose-dependent. In the LPS-induced and ConA-induced proliferation, LP1 at the tested doses significantly increased splenic lymphocyte proliferation, indicating that B- and T-lymphocytes may be the target cells for LP1.

## 3. Experimental Section

### 3.1. Materials

The human ovarian cancer cell lines, SKOV3 and HO8910, were supplied by the cell bank of the Chinese Academy of Sciences (Shanghai, China). SPF BALB/c male mice were provided by the experimental animal center of Guangxi Medical University (Nanning, China). PBS buffer was obtained from Wuhan Boster Bio-Engineering Co., Ltd. (Wuhan, China). Sodium chloride for injection was obtained from Kunming Yusi Pharmaceutical Co., Ltd. (Kunming, China). RPMI-1640 with an improved nutrient solution was purchased from Thermo Fisher Scientific (Beijing) Inc. (Beijing, China). Sword bean protein and lipopolysaccharide were purchased from Beijing Bo Ao Tuoda Technology Co., Ltd. (Beijing, China). 3-(4,5-Dimethylthiazol-2-yl)-2,5-diphenyltetrazolium bromide (MTT) and 0.25% trypsin (containing EDTA) were purchased from Sigma Chemicals Co., Perth, ND, USA. All other chemicals and solvents were analytical grade and used without further purification.

### 3.2. Isolation and Purification of Polysaccharides

First, the dried longan pulp (2.0 kg) was extracted with distilled water at 90 °C for 4 h (three times) and then filtered with cotton gauze. The extracted solutions were combined and concentrated by rotary evaporation under reduced pressure below 65 °C and subjected to the Sevag method [49] to remove free proteins. The crude polysaccharide thus obtained was labeled LP. Then, the LP extract (2.00 g) was further purified by dissolution in distilled water and application to a column (Φ2.5 cm × 80 cm) of DEAE-cellulose. After the sample was loaded, the column was successively washed with distilled water, 0.125 M sodium chloride and 0.30 M sodium hydroxide aqueous solution at an elution rate of 0.4 mL/min. An improved phenol-sulfuric acid detector was used online, and the eluate was collected automatically [50]. A total of four fractions of polysaccharide were obtained and labeled F-1, F-2, F-3 and F-4. Third, the largest NaCl-eluted fraction, F-1, was collected, dialyzed for 2 days, concentrated and lyophilized. The F-1 fraction was further purified by dissolution in distilled water using ultrasound and subjected to Sephacryl S-300 HR gel filtration chromatography (Φ2.0 cm × 50 cm) (Shanghai Stars Biological Technology Co., Ltd., Shanghai, China) after filtration with a 0.45-μm microporous membrane filter. The loading sample was washed with distilled water at an elution rate of 0.75 mL/min and monitored with a phenol-sulfuric acid detector. The purified fraction containing carbohydrate was collected, concentrated, precipitated with alcohol and dried under vacuum to yield a sample of purified polysaccharide, which was labeled LP1.

### 3.3. HPGPC to Determine the Molecular Weight of the Polysaccharide

The preparation of standard solutions and sample solutions was performed as follows: dextran 40, a series of standard glucans (molecular weights 5900, 11,800, 22,800, 47,300, 112,000, 212,000 and 404,000) and dried longan polysaccharide LP1 were accurately weighed; ultrapure water was then added, and the samples were dissolved with the aid of ultrasonication over 15 min. The final concentration of the standard solutions was 10 mg/mL, and the solutions were filtered with a 0.45-μm microporous filter.

The chromatographic conditions were as follows: a chromatographic column (TSK-4000PW, Φ7.5 mm × 300 mm, Tosoh Bioscience Shanghai Co., Ltd., Shanghai, China), a gel column (TSK-3000SW, Φ7.5 mm × 300 mm, Tosoh Bioscience Shanghai Co., Ltd.) and a guard column (TSK-Guard SW, Φ7.5 mm × 75 mm, Tosoh Bioscience Shanghai Co., Ltd.) were employed; the two gel columns and the guard column were used in series. The chromatographic column temperature was 23 °C with a flow velocity of 1 mL/min; detection was performed using a refractive index detector (Agilent G1362A, Agilent Technologies (China) Co., Ltd., Beijing, China). A volume of 20 μL each of the standard sample and LP1 were injected separately. The mobile phase was ultrapure water. The evolution volume (*V*_e_) was measured, and through data analysis, (Agilent GPC software, Beijing, China), the standard curve was drawn with the *V*_e_ as the abscissa and the logarithm of the *M*_r_ as the vertical axis.

### 3.4. Monosaccharide Composition

The LP1 polysaccharide sample (250.0 mg) was hydrolyzed with 5 mL of 1.0 mol/L TFA at 102 °C for 6 h in a sealed ampule, cooled to room temperature, transferred to a 10-mL volumetric flask and then neutralized with a 3-mol/L NaOH solution until the pH reached 7.0. The sample was then diluted with distilled water, mixed thoroughly and then centrifuged for 10 min at 4000 rpm. The supernatant was collected, precisely measured and mixed with seven different monosaccharide solutions (mannose, rhamnose, galacturonic acid, glucose, galactose, xylose and arabinose); each solution contained 0.4 mL at 0.2 mg/mL in two test tubes. Next, 0.4 mL of a 0.5 mol/L 1-phenyl-3-methyl-5-pyrazolone (PMP) solution in methanol and 0.4 mL of a 0.4 mol/L NaOH solution were sequentially added. The solutions were mixed, heated in a 70 °C water bath for 100 min and then cooled to room temperature. This procedure was followed by neutralization with 0.5 mL of a 0.3-mol/L HCl solution, the addition of distilled water to a volume of 2.0 mL and extraction with 5.0 mL chloroform. The upper aqueous phase was then filtered through a 0.45-μm microporous membrane.

Chromatography was performed with an Agilent TC-C18 column (Φ4.6 mm × 250 mm, 5 μm) (Agilent Technologies (China) Co., Ltd.) under the following conditions: column temperature: 25 °C; UV detection: 250 nm, with a PD-10-a VP Plus detector (Agilent Technologies (China) Co., Ltd.); sample quantity: 10 μL; mobile phase: 80 mL of 0.05 mol/L ammonium acetate solution and 20 mL of acetonitrile; flow rate: 1.0 mL/min; and elution time: 35 min.

### 3.5. Characterization

LP1 was placed in a 5-mm NMR tube and dissolved in D_2_O (0.5 mL). The NMR spectra were recorded with a Bruker AVIII 600 NMR spectrometer (Bruker (Beijing) Scientific Technology Co., Ltd., Beijing, China) at room temperature. All chemical shifts are reported in parts per million relative to the internal standard TSP-d4. Infrared spectroscopy of the samples was performed with a Nicolet 170SX FT-IR spectrometer from 400–4000 cm^−1^ with a DGTS detector and DMNIC 3.2 software (Thermo Fisher Scientific (Beijing) Inc., Beijing, China). Gas chromatography (GC) of the alditol acetate derivatives of the saccharides, as reported in the literature [51], was carried out on an HP 6890 instrument (Hewlett Packard, Palo Alto, CA, USA) with a DB-225 column (Φ0.25 mm × 15 m) (Agilent Technologies (China) Co., Ltd.) from 180 to 220 °C at 4 °C/min.

### 3.6. Biological Assays

#### 3.6.1. Anti-Tumor Activity *in Vitro*

##### 3.6.1.1. Cell Lines and Culture

Human gastric carcinoma SGC-7901 cells were maintained in RPMI-1640 medium supplemented with 10% FBS, penicillin (100 U/mL) and streptomycin (100 mg/L) at 37 °C in a humidified atmosphere with 5% CO_2_. The culture was passaged every 2 or 3 days. The final cell concentration was 3 × 10^7^ cells/mL.

##### 3.6.1.2. Assay of the Inhibition of Tumor Cell Proliferation *in Vitro*

The inhibitory effects of LP1 on the proliferation of SKOV3 and HO8910 cells (3 × 10^7^ cells/mL) were determined *in vitro* with the colorimetric MTT assay [52]. The doses of LP1 applied to the SKOV3 tumor cells were 5, 10, 20 and 40 mg/L. The doses of LP1 applied to the HO8910 tumor cells were 40, 80, 160 and 320 mg/L. The concentration of 5-fluorouracil used for the positive control was 5 mg/L. The absorbance was measured at a wavelength of 570 nm with a 96-well microplate ELISA reader (Bio-Tek Instruments, Inc., Winooski, VT, USA). All experiments were performed in triplicate. The inhibition percentage of tumor cell proliferation was calculated according to the formula below:

(1) $$\text{Inhibition ratio }(\%)=\left(1-\frac{OD}{OD_{0}}\right)\times 100$$

where *OD* and *OD*_0_ are the absorbances of the treated cells and the untreated cells, respectively.

##### 3.6.1.3. Effect of LP1 on the Proliferation of Macrophages *in Vitro*

Isolation of peritoneal macrophages. Macrophages were prepared from BALB/c mice, as described previously [53]. Briefly, peritoneal macrophages were harvested from 2 to 3 BALB/c mice that had been intraperitoneally injected with 3 mL of thioglycollate for three days before a sterile peritoneal lavage with 10 mL of Hank’s balanced salt solution. After centrifugation at 1000 rpm/min for 5 min, the cell pellets were suspended in RPMI-1640 supplemented with 10% FBS, penicillin (100 IU/mL) and streptomycin (100 μg/mL), seeded in a 96-well plate at a cell density of 2 × 10^6^ cells/mL and allowed to adhere for 3 h at 37 °C in a 5% CO_2_ humidified incubator. Non-adherent cells were removed by thorough washing, leaving monolayers of adherent cells consisting of ~99% macrophages. The viability of the adherent cells was assessed by the trypan blue exclusion test, and the proportion of macrophages was determined via cell morphology using a microscope (>95%).

Phagocytic assay with the neutral red method. [54] The phagocytic ability of the macrophages was measured via neutral red uptake. The cells were cultured with LP1 (0, 25, 50 and 100 μg/mL) or LPS (10 μg/mL) at 37 °C and 5% CO_2_ for 48 h. the culture media were removed, and 100 μL/well of 0.075% neutral red were added; the cells were then incubated in 96-well plates for 4 h. The supernatant was discarded, and the cells were washed twice with PBS to remove the neutral red that was not phagocytized by the macrophages. Cell lysis buffer (ethanol and 0.01% acetic acid at a ratio of 1:1, 100 μL/well) was added to lyse the cells. After the cells were incubated at room temperature overnight, the optical density at 540 nm was measured with a microplate reader.

Nitric oxide assay. Nitrite accumulation in the medium was used as an indicator of NO production as previously described [55], and colorimetric assays [56] with the Griess reagent were used to detect NO levels (Nanjing SunShine Biotechnology Co., Ltd., Nanjing, China). Adherent macrophages were cultured with various concentrations of LP1 (0, 25, 50 and 100 μg/mL) or LPS (10 μg/mL) at 37 °C for 48 h. At the end of the culture period, 100 μL of the isolated supernatant was allowed to react with the Griess reagent (1% sulfanilamide, 0.1% naphthylethylenediamine dihydrochloride and 2.5% H_3_PO_4_) at room temperature for 10 min. Nitrite production was determined by comparing the absorbance at 550 nm with a standard curve generated using NaNO_2_.

Cytokine assays. [57] Adherent macrophages were cultured with various concentrations of LP1 (0, 25, 50 and 100 μg/mL) or LPS (10 μg/mL) at 37 °C for 24 h. At the end of the culture period, the supernatants were collected and the level of the cytokines, TNF-α, IL-6 and IL-1β, were determined with ELISA kits (Guangxi Nanning Chujie Biological Technology Co., Ltd., Nanning, China), according to the manufacturer’s instructions.

#### 3.6.2. Animal Immune Experiments

##### 3.6.2.1. Chemicals and Animals

Kunming mice (SPF), weighing 20 ± 2 g and 6–8 weeks old, were provided by the Center of Animal Experimentation of Guangxi Medical University (Nanning, China) (experimental animal use license: SYKG Guangxi 2003-0005; laboratory animal production license: SCXKG Guangxi 2003-2003). CY for injection (No. 04120705) was purchased from Shanxi PuDe Pharmaceutical Co., Ltd. (Shanxi, China). The mouse IFN-γ ELISA kit (No. 978941214) and mouse IL-2 ELISA kit (No. 1208911214) were provided by Wuhan Boster Bio-Engineering Co. Ltd. (Wuhan, China). Neutral red (No. E895), concanavalin A (No. c-2010) and LPS (No. L2880), as a B-cell mitogen, were purchased from Beijing Bo Ao Tuoda Technology Co., Ltd. (Beijing, China). MTT (No. 20110613) and 0.25% trypsin (with EDTA) were purchased from Solarbio (Beijing, China).

##### 3.6.2.2. Cyclophosphamide-Induced Immunosuppression Mouse Model

After one day of adaptability feeding, the mice were randomly allocated to a blank group, a cyclophosphamide (CY) inhibition group and an LP1 group, according to weight. Ten mice were in each experimental group (half male and half female), including 3 groups with different doses of LP1: CY + LP1 (I) (320 mg/kg), CY + LP1 (II) (160 mg/kg) and CY + LP1 (III) (80 mg/kg). The mice in the blank control group were given an intraperitoneal injection of saline. The mice in the inhibition group and the experimental group were given an intraperitoneal injection of cyclophosphamide once every three days at a dose of 20 mg/kg and a capacity of 10 mL/kg. Administration was intragastric. The blank control group was given the same amount of physiological saline, with a capacity of 10 mL/kg, once per day over a period of 21 days [58,59].

##### 3.6.2.3. Measurement of IL-2 and IFN-γ

Blood was taken from the eyeballs of the mice and put into a 2 mL EP tube. It was then centrifuged for 10 min at 3500 rpm/min at 4 °C. The upper, clarified serum was drawn and the amount of IL-2 and IFN-γ in the serum was measured via ELISA [60].

##### 3.6.2.4. Macrophage Phagocytosis Assay

Macrophages were prepared from BALB/c mice as described previously [61]. After removing the eyeballs and soaking the mice with 75% ethanol for 2 min, 5 mL sterile PBS solution was injected into the celiac region of the mice, followed by kneading for 2 min. The peritoneal fluid was then removed with a syringe (while avoiding the abdominal viscera, which could result in bleeding), and the abdominal fluid was centrifuged at 2000 rpm/min for 15 min. The centrifugal supernatant was discarded, and PRMI-1640 with 10% fetal bovine serum (FBS) was then added to the cell pellet. The cells were dispersed by gentle trituration. The cells were seeded at a density of 1 × 10^6^ cells/mL in a 96-well plate, with each well containing 100 μL of suspension, and were incubated at 37 °C in a humidified 5% CO_2_ atmosphere for 4 h. All cell culture solutions were then discarded, and the non-adherent cells were removed by washing. To each well, 100 μL 0.1% neutral red dye was then added (0.2500 g neutral red in 250 mL 0.9% saline), and the plates were incubated at 37 °C in a 5% CO_2_ incubator for another 30 min. Excess neutral red solution was discarded. The cells in each well were washed with PBS solution three times, and 200 μL of a cell lysis solution was added (acetic acid:anhydrous ethanol, 1:1). The plate was gently oscillated and then placed in a 4 °C environment overnight. The optical density at 570 nm was measured for each well with an enzyme marking instrument.

##### 3.6.2.5. Analysis of B- and T-Cell Proliferation

The spleen was weighed, crushed into 1-mm^3^ pieces and digested at 37 °C with 0.25% pancreatic enzymes. PRMI-1640 containing 10% calf serum was then added at twice the volume, followed by gentle tapping to mix and render the pancreatic enzymes inactive. This action was performed to reduce the influence of the pancreatic enzymes on the living cells. The material was then passed through a 200-mesh cellular sieve, filtered and centrifuged for 8 min at 1000 rpm/min. The supernatant was discarded, leaving the pellet, which was composed of spleen cells. PRMI-1640 culture medium was then added to prepare a single spleen cell suspension. One hundred microliters of the spleen cell suspension were placed into each well of a 96-well plate at a concentration of 2 × 10^6^ cells/mL, and then, 100 μL LPS (30 μg/mL) was added to each well as the B-lymphocyte transformation irritant. This was repeated for each sample in three wells. The plates were incubated at 37 °C in a 5% CO_2_ for 66 h; 20 μL of MTT was then added to each well followed by incubation at 37 °C for another 4 h. The supernatants were discarded; 150 μL of dimethyl sulfoxide (DMSO) was then added, and the plates were oscillated. The *A*_490_ was recorded as an index of stimulating the proliferation of the B-cells in an enzyme marking instrument. The value for the experiments that did not involve stimulation with LPS was set at 100%, and the values for the other experiments were converted into a percentage expression. A statistical analysis was performed. For monitoring the proliferation of T-cells, the procedure was the same as that described for B-lymphocyte cell proliferation above, except that concanavalin A (ConA) replaced LPS for the stimulation [62].

### 3.7. Statistics

All the experiments were performed at least in duplicate, and the analyses of all the samples were run in triplicate and averaged. The statistical analysis utilized SPSS 13.0 software (SPSS Inc., Chicago, IL, USA) for processing. The results are presented as the means of three determinations, *χ̄* ± SD. The results were analyzed using a one-way analysis of variance (ANOVA) for the mean differences among the samples. A *p*-value <0.05 was considered to be statistically significant.

## 4. Conclusions

The water-soluble polysaccharide LP1 was successfully isolated from the *Dimocarpus longan* pulp, purified and partially characterized. LP1 contained α- and β-d-glucans. The apparent mean *M*_r_ of the sample was 1.1 × 10^5^ Da. The sample exhibited a significant inhibition of the growth of SKOV3 and HO8910 cells, with the inhibition percentage of HO8910 cells exceeding 50%. LP1 also stimulated spleen lymphocyte proliferation and macrophage function in a dose-dependent manner. The experimental results suggest that LP1 has significant potential as a safe and effective reagent to prevent the development of tumor cells by killing the tumor cells directly and, more importantly, by improving immunocompetence, as observed in the *in vitro* and murine experiments, respectively. Further investigation of LP1 is important and will be reported elsewhere, as no medicine currently exists that can effectively treat cancer without side effects.

## Figures and Tables

**Table 1.** ^1^H NMR and ^13^C NMR shifts of LP1 and their assignments.

Glycosyl residues	H-1/C-1	H-2/C-2	H-3/C-3	H-4/C-4	H-5/C-5	H-6a, H-6b/C-6
→4)-α-d-Glcp-(1→	5.27/92.11	3.42/70.99	3.56/72.85	3.89/73.84	3.74/75.81	3.66,3.64/60.65
	5.09/92.00	3.40/70.99	3.56/72.85	3.87/74.24	3.77/72.46	3.67, 3.57/60.55
→4,6)-β-d-Glcp-(1→	4.51/95.79	3.12/73.78	3.34/75.51	3.75/72.12	3.58/72.46	3.86,3.92/63.28
→2)-β-d-Fruf-(1→	3.56,3.74/63.78	–/103.59	4.07/76.23	3.92/74.57	3.74/81.27	3.64,3.59/62.99
→2)-l-sorbose-(1→	3.56,3.66/64.04	–/98.03	3.70/70.99	3.56/74.33	3.52/71.03	3.75,3.59/62.35

**Table 2.** HMBC data and assignments of LP1.

Glycosyl residues	δH/δC atom	Observed connectivities

		δH/δC atom
A→4)-α-d-Glcp-(1→	5.27 A-H1	72.12 B-C4
A′→4)-α-d-Glcp-(1→	5.09 A′-H1	71.11
B→4,6)-β-d-Glcp-(1→	4.51 B-H1	–
C→2)-β-d-Fruf-(1→	103.41 C-C2	3.56 D-H1
D→2)-l-sorbose-(1→	98.03 D-C2	3.56 D-H3 3.86 B-H6

**Table 3.** Cytotoxicity of LP1 in SKOV3 tumor cells after 48 h (*χ̄* ± SD, *n* = 6). *χ̄* ± SD: the means of six determinations.

Group	Concentration (mg/L)	*A*_570 nm_	Inhibition (%)
Control	–	0.799 ± 0.005	0
5-Fluorouracil (5-FU)	5	0.350 ± 0.018 **	56.2
LP1	40	0.480 ± 0.017 **	39.9
	20	0.494 ± 0.007 **	38.2
	10	0.496 ± 0.005 **	37.9
	5	0.504 ± 0.002 **	36.9

*Versus* the control group:

** *p* < 0.01.

**Table 4.** Cytotoxicity of LP1 in HO8910 tumor cells after 72 h (*χ̄* ± SD, *n* = 6).

Group	Concentration (mg/L)	*A*_570 nm_	Inhibition (%)
Control	–	0.610 ± 0.006	0
5-FU	5	0.284 ± 0.007 **	53.4
LP1	320	0.303 ± 0.008 **	50.3
	160	0.328 ± 0.004 **	46.2
	80	0.330 ± 0.006 **	45.9
	40	0.355 ± 0.008 **	41.8

*Versus* the control group:

** *p* < 0.01.

**Table 5.** Effect of LP1 on macrophage phagocytic ability *in vitro* (*χ̄* ± SD, *n* = 6). LPS, lipopolysaccharide.

Group	Concentration (μg/mL)	Phagocytic count (OD_570 nm_)
Blank control group	–	0.184 ± 0.0071
LPS-positive group	10	0.306 ± 0.0167 *
LP1	25	0.293 ± 0.0128 *
	50	0.312 ± 0.0132 *
	100	0.329 ± 0.0097 *

* Compared with the normal control, *p* < 0.05.

**Table 6.** Effect of LP1 on NO production by mouse peritoneal macrophages (*χ̄* ± SD, *n* = 6).

Group	Concentration (μg/mL)	NO production (μM)
Blank control group	–	7.51 ± 0.781
LPS-positive group	10	25.05 ± 1.082 *
LP1	25	18.85 ± 1.012 *
	50	20.75 ± 0.976 *
	100	22.85 ± 0.998 *

* Compared with the normal control, *p* < 0.05.

**Table 7.** Effect of LP1 on TNF-α secretion by mouse peritoneal macrophages (*χ̄* ± SD, *n* = 6).

Group	Concentration (μg/mL)	TNF-α concentration (pg/mL)
Blank control group	–	172.85 ± 17.09
LPS-positive group	10	560.65 ± 34.31 *
LP1	25	427.65 ± 22.64 *
	50	451.22 ± 26.98 *
	100	497.65 ± 24.46 *

* Compared with the normal control, *p* < 0.05.

**Table 8.** Effect of LP1 on IL-6 secretion by mouse peritoneal macrophages (*χ̄* ± SD, *n* = 6).

Group	Concentration (μg/mL)	IL-6 concentration (pg/mL)
Blank control group	–	20.13 ± 1.69
LPS-positive group	10	59.55 ± 4.19 *
LP1	25	68.65 ± 3.64 #
	50	72.92 ± 4.08 #
	100	78.85 ± 3.86 #

* Compared with the normal control, *p* < 0.05;

# compared with the LPS-positive control, *p* < 0.05.

**Table 9.** Effect of LP1 on IL-1β secretion by mouse peritoneal macrophages (*χ̄* ± SD, *n* = 6).

Group	Concentration (μg/mL)	IL-1β concentration (pg/mL)
Blank control group	–	34.51 ± 2.89
LPS-positive group	10	64.35 ± 4.04 *
LP1	25	54.87 ± 3.12 *
	50	58.17 ± 2.88 *
	100	64.14 ± 3.49 *

* Compared with the normal control, *p* < 0.05.

**Table 10.** Effect of the LP1 polysaccharide from *Dimocarpus longan* pulp on cyclophosphamide (CY)-induced immunosuppression of mouse splenic B-cell proliferation (*χ̄* ± SD, *n* = 8).

Group	*A*_570 nm_		Stimulus index 100%

	LPS stimulated	No LPS stimulation	
Control	0.321 ± 0.08	0.276 ± 0.10	116
CY	0.243 ± 0.03	0.226 ± 0.13	108
CY + LP1 (I)	0.932 ± 0.34 *	0.637 ± 0.15	146
CY + LP1 (II)	0.856 ± 0.35 *	0.681 ± 0.08	126
CY + LP1 (III)	0.810 ± 0.24 *	0.673 ± 0.15	120

* Compared with CY, *p* < 0.05.

**Table 11.** Effect of the LP1 polysaccharide from *Dimocarpus longan* pulp on cyclophosphamide (CY)-induced immunosuppression of mouse splenic T-cell proliferation (*χ̄* ± SD, *n* = 8).

Group	*A*_570 nm_		Stimulus index 100%

	ConA stimulated	No ConA stimulation	
Control	0.358 ± 0.05	0.276 ± 0.10	130
CY	0.263 ± 0.02	0.226 ± 0.13	116
CY + LP1 (I)	0.914 ± 0.13 *	0.637 ± 0.15	143
CY + LP1 (II)	0.849 ± 0.28 *	0.681 ± 0.08	125
CY + LP1 (III)	0.804 ± 0.21 *	0.673 ± 0.15	119

* Compared with CY, *p* < 0.05.
